# Patient and Public Involvement (PPI) in Secondary Data Analysis: Protocol for a Targeted Review

**DOI:** 10.1111/hex.70532

**Published:** 2025-12-18

**Authors:** Jonathan Broomfield, Elizabeth Fisher, Eleanor Booth, Samina Begum, Cecily Henry, Julie Roberts, Laura J. Gray, PPI‐SMART Team

**Affiliations:** ^1^ Division of Public Health and Epidemiology University of Leicester Leicester UK; ^2^ Division of Respiratory Sciences University of Leicester Leicester UK; ^3^ University of Leeds Leeds UK; ^4^ Public Contributor UK; ^5^ NIHR Applied Research Collaboration East Midlands University of Leicester Leicester UK; ^6^ NIHR Leicester Biomedical Research Centre University of Leicester Leicester UK; ^7^ Leicester British Heart Foundation Centre of Research Excellence University of Leicester Leicester UK

## Abstract

**Introduction:**

Patient and public involvement (PPI) is increasingly recognised as a vital component of health research, promoting inclusivity, relevance and impact. While PPI is well‐integrated into primary research, its role in secondary data analysis, where researchers work with pre‐existing data, remains underexplored. This targeted review aims to assess the prevalence, characteristics, and evolution of PPI in secondary data analysis studies, with a focus on articles published in The British Medical Journal (The BMJ) across selected years.

**Methods and Analysis:**

This targeted literature review will examine original research articles published in *The BMJ* in 2014, 2019, and 2024. These years were selected to capture trends before and after the journal's 2015 mandate requiring PPI statements. Studies will be included if they utilise secondary data to address original research questions. A comprehensive search strategy using PubMed and a defined set of keywords related to secondary data will be employed. Articles will be screened independently by two reviewers at both title/abstract and full‐text stages. Data extraction will focus on study characteristics, PPI inclusion and outcomes influenced by PPI. Quantitative analysis will assess the prevalence and trends of PPI, while qualitative description of PPI statements will be used to summarise current practice and identify successful, effective practices. A representative sample of reviewed PPI statements will be shared in text and audio formats with six public contributors, who will assess them using a Likert scale and provide feedback on good and poor PPI practices during a facilitated meeting.

**Ethics and Dissemination:**

As this review involves only published literature, ethical approval is not required. Findings will be disseminated through peer‐reviewed publication, academic conferences and community engagement events to inform best practices and promote meaningful PPI in secondary data research.

**Patient and Public Involvement:**

Six public contributors will be involved in the review process, ranking PPI statements and providing feedback on the level of PPI input to the studies as well as the level of reporting in the articles (detailed in Section 3.4). Additionally, two public contributors reviewed and are co‐authors on this protocol, and we will invite another three public contributors to review any subsequent publications or materials produced by this review. We will adhere to the UK Standards for Public Involvement, ensuring we provide adequate support and inclusive opportunities for public contributors (e.g., by sending materials in text and audio formats in advance of meetings) and including them as co‐authors where appropriate to amplify their voices and maximise their impact on the project. Public contributors will be reimbursed in accordance with NIHR INVOLVE guidelines.

## 1 Introduction

Patient and public involvement (PPI) primarily concerns research being conducted ‘with’ or ‘by’ patients and members of the public rather than ‘to’, ‘about’ or ‘for’ them [1, 2]. It is being increasingly recognised as essential in health research [3], leading to more inclusive and impactful results [4, 5] as well as empowerment of PPI contributors. Funders such as the NIHR now mandate that grant and fellowship applications include details of how working with people and communities has informed applications and who has been involved and why. Additionally, some journals (such as The British Medical Journal [*The BMJ*] in 2015 and *BMJ Open* in 2018 [6]) now require a PPI statement to accompany articles, explaining whether PPI was deemed relevant and how it was conducted.

The GRIPP2 statement (Guidance for Reporting Involvement of Patients and the Public) was published in *The BMJ* in 2017 [7], and contains a revised set of guidelines designed to improve how researchers report PPI in health and social care studies. Available in both short‐form and long‐form formats, it promotes transparency, consistency and quality in reporting, helping others understand the impact of PPI and learn from best practices. Researchers submitting articles to *The BMJ* for publication are encouraged to consult the GRIPP2 short‐form reporting guidelines, and provide in their PPI statement a response to the following [8]:

- At what stage in the research process were patients/public first involved in the research and how?
- How were the research question(s) and outcome measures developed and informed by their priorities, experience and preferences?
- How were patients/public involved in the design of this study?
- How were they involved in the recruitment to and conduct of the study?
- Were they asked to assess the burden of the intervention and time required to participate in the research?

*The BMJ* provides successful examples of PPI in fields of health research such as randomised controlled trials, feasibility studies and prospective observational studies [8]. However, PPI integration into secondary data analysis, where researchers work with pre‐existing datasets, remains limited and underexplored [9]. Common types of secondary analysis include re‐analysis of clinical trial data, subgroup analyses and analyses of routinely collected electronic health records or pre‐existing survey datasets. These approaches are crucial in health care research because they maximise the value of existing data and can generate insights more efficiently and cost‐effectively than primary data collection. They are particularly important for exploring long‐term outcomes, rare events and real‐world effectiveness across diverse patient populations. Involving patients and the public can help ensure the questions addressed are relevant and the findings are interpreted in ways that reflect lived experience. Possible methods of inclusion for PPI in secondary data analysis have been suggested at the design, analysis and interpretation stages [9].

This protocol proposes a targeted review to assess the prevalence, characteristics and evolution of PPI in secondary data analysis studies. Understanding how PPI is currently implemented will help guide future research practices and promote inclusive methodologies. A targeted review will enable an overview of current practice to motivate more comprehensive future research. The protocol is reported in line with the Preferred Reporting Items for Systematic Reviews‐Protocols (PRISMA‐P) guidelines [10] where appropriate, although as it is not a systematic review it has not been registered on PROSPERO.

## 2 Objectives

1. Investigate how many studies conducting secondary data analysis include PPI as part of their study.
2. Investigate the reasons for not conducting PPI for secondary data analysis.
3. Identify details of case studies and themes of inclusion and exclusion for PPI in statistical analyses of secondary data.
4. Identify changes in the last decade of PPI in secondary data analysis, during which time PPI reporting has become mandatory in some journals.
5. Identify good practice in PPI in secondary data analysis to inform future research, including the prevalence of training being provided for PPI members.

## 3 Methods

### 3.1 Study Design

This is a targeted review of published literature on PPI in secondary data analysis. Specifically, we will focus on articles published in *The BMJ* (not including partner journals such as *BMJ Open* or *BMJ Global Health*). *The BMJ* has required a PPI statement to be reported since 2015 [6]. The review will include both quantitative and qualitative analysis and will be reported according to PRISMA guidelines. The study team consists of researchers, with experience of quantitative analysis as well as basic qualitative analysis when considering PPI in statistical research [11], and public contributors.

### 3.2 Eligibility Criteria

We will only include studies published in *The BMJ*, excluding partner journals. We will include articles published in 2014, 2019 and 2024 to allow us to investigate trends in conducting and reporting PPI over time. These years were selected as a PPI statement has been required in all research articles published in *The BMJ* since 2015; thus, results from 2014 will give an indication of the conducting of PPI in secondary data analysis before a statement was mandatory, results from 2019 will be representative of a few years of requiring a PPI statement and results from 2024 of 10 years after it was mandatory. We pragmatically selected three publication years to enable us to assess changes over a 10‐year period without the need to review every year (which would likely yield similar results), balancing comprehensiveness with feasibility. We focused exclusively on The BMJ as different journals have mandated PPI statements at different times, making standardised comparisons challenging. Additionally, the BMJ is a generic journal accepting research articles across a range of medical fields rather than specialising in a particular discipline, making the results more generalisable. Further, while the PPI statement is mandatory, conducting PPI is not mandatory for articles included in *The BMJ*, allowing our review to evaluate current conduct of PPI in secondary data analysis.

Studies will be included if they use secondary data in their analysis to answer an original research question, by which we mean any form of existing data that was not originally collected to answer that research question. This includes (but is not limited to) research using data from routinely collected electronic health records, biobanks, existing research data or cohort studies, disease‐specific registries, re‐analysis of clinical trial data, and re‐analysis of survey data. Studies that use new data collected specifically to answer the research question in that study will be excluded, as will studies not conducting any original research. We will also not include evidence synthesis studies, even if individual patient data have been included.

### 3.3 Search Strategy

PubMed will be used to filter by journal (“BMJ”[Journal]) and year of publication (for 2024, “2024/01/01”[Date ‐ Publication]: “2024/12/31”[Date ‐ Publication] – equivalents for 2019 and 2014 will be used). To identify articles conducting secondary data analysis, the following search terms have been defined (using a combination of statistical and clinical experience, discussion with a library research services consultant and AI tools):

("secondary data" OR "secondary analysis" OR "pre‐existing data" OR "preexisting data" OR "reanalysis" OR "re‐analysis" OR "administrative data" OR "claims data" OR "insurance claims" OR "linked data" OR "data linkage" OR "real‐world data" OR "RWD" OR "real‐world evidence" OR "RWE" OR "population health data" OR "healthcare utilisation data" OR "healthcare utilization data" OR "observational data" OR "longitudinal data" OR "surveillance data" OR "retrospective" OR "cohort study" OR "primary care database" OR "target trial emulation" OR "big data" OR "health outcomes" OR "data mining" OR "routinely collected data" OR "routine data" OR "electronic healthcare record" OR "EHR" OR "electronic medical record" OR "EMR" OR "registry data" OR "health information system" OR "clinical database" OR "clinical data repository" OR "existing data" OR "hospital episode statistics" OR "HES" OR "biobank data" OR "clinical practice research datalink" OR "CPRD" OR "SAIL" OR "secure anonymised information linkage")

As this is a targeted review, other journals, grey literature and reference lists of included studies will not be included in the search strategy.

Following the search, identified articles will have their titles, abstracts and publication details reviewed to exclude papers not published in 2014, 2019 or 2024, papers not published in *The BMJ* and papers that are not original research articles. This will be done in Rayyan [12] by a team of three reviewers, with each reviewer being randomly assigned a number of articles such that each article will be independently reviewed by two reviewers. Any conflicts between two reviewers will be resolved blindly by the third reviewer. Remaining papers will have the full text screened to determine whether any secondary data analysis was conducted. This will be done by a team of six reviewers, with each article reviewed independently by two reviewers and conflicts resolved blindly by a seventh reviewer. After this stage, any remaining studies are eligible for inclusion in the targeted review and will have their PPI statements reviewed. The included studies will be exported to Excel for management and classification of PPI statements, where details of the PPI inclusion, adherence to the GRIPP2 short‐form domains [7] or reasons for lack of inclusion will be noted.

We do not anticipate a risk of bias in the individual studies identified but recognise that articles published in *The BMJ* since 2015 are more likely to incorporate PPI in their research (given the mandatory PPI statement) than articles published in similar journals (that do not require such a statement).

### 3.4 Data Extraction and Analysis

Data will be extracted on study characteristics: publication year, country that study was carried out, the source/type of data used, and type and extent of PPI, including outcomes and stages of the research influenced by PPI (such as study design, outcome definition, interpretation of results and drafting of the manuscript). We will investigate any reasons for not undertaking PPI and identify reported PPI activities that do not in fact constitute PPI, such as stakeholder engagement. The number of papers reporting (a) a PPI statement and (b) PPI being conducted as part of the research will be analysed to determine prevalence and trends over time. Adherence to the required responses for *The BMJ* (detailed in 5) will be assessed. A qualitative descriptive approach [13, 14] will be used to summarise the content of the PPI statements. We will explore whether PPI was involved in the design of the research study, analysis of the data, wider aspects of the project or in engagement only. If PPI was conducted but reported in insufficient detail to determine this, we will contact corresponding authors.

Data extraction of PPI statements that do conduct PPI will be structured using the GRIPP2 short‐form reporting checklist [7]. Each statement will be mapped against the five domains: aims, methods, study results, discussion/conclusions and reflections/critical perspective. Reported information will be coded to the relevant domain, recorded as gaps where no information is provided for a domain. This framework will enable us to assess which domains are most and least frequently reported and to describe the nature of PPI involvement when it is conducted, as well as identify examples of good practice and areas where reporting remains limited.

We will meet with six public contributors where we will share examples of PPI statements and ask for feedback regarding good and bad PPI practice. Examples will be selected to engage public contributors with key aspects of the draft analysis and areas of uncertainty. To facilitate this, we will send the PPI statements in text and audio‐recorded formats to the contributors beforehand and will ask them at the meeting to rate the statements on a Likert scale and provide additional comments if desired. This approach will provide perspectives on what is considered good PPI practice by researchers and by public contributors and highlight if there is any disparity between the two.

To show the fields over which we will report findings of PPI conduct in secondary data analysis and how statements of studies including PPI will be mapped to the GRIPP2 short‐form domains, we have included an example table in the Supporting Information. This also includes details of the extraction template that we will adopt. Details of the protocol and planned targeted review have been pre‐registered with Open Science Framework [15].

### 3.5 Patient and Public Involvement

Six public contributors for this review were recruited from the PPI‐SMART group [16], an existing group of public contributors with prior experience in methodological and secondary data analysis. Recruitment was based on contributors’ interest in methods‐focused project. The group is diverse in terms of geography, age, sex and ethnicity, ensuring a range of perspectives. To support equity and accessibility, all materials will be provided in advance of the meeting in both text and audio formats, and PPI members will be offered one‐to‐one calls to discuss any questions ahead of meeting. While formal training is not required due to their prior experience, orientation and support will be provided to ensure clarity and confidence in completing the rating and discussion activities.

## 4 Conclusion

This targeted review builds on existing literature that has highlighted both the importance and challenges of embedding PPI in healthcare research. Previous research has demonstrated that PPI can improve the relevance and impact of research findings [3, 4, 5], yet its integration into secondary data analysis remains limited [9]. The introduction of mandatory PPI statements in journals such as *The BMJ* [6] and *BMJ Open* [8] and development of reporting checklists such as GRIPP2 [7] reflects a growing recognition of the need for transparency and accountability in reporting involvement.

Our review will provide a comprehensive assessment of how PPI is integrated into secondary data analysis studies published in *The BMJ* over a 10‐year period. By examining the prevalence, nature and evolution of PPI practices, the review will generate valuable insights into how researchers engage with patients and the public when working with pre‐existing datasets. The mixed‐methods approach will highlight best practices and identify gaps and opportunities for more meaningful involvement.

The anticipated outcomes include a clearer understanding of the extent to which PPI is embedded in secondary data research, illustrative case studies of effective involvement and evidence of how practices have changed in response to policy shifts such as *The BMJ*'s PPI statement requirement. These findings will inform future research by guiding methodological approaches and supporting the development of tailored PPI strategies for secondary data contexts. The specific outputs will be a publication detailing full results of the review, presentations at academic conferences and a plain English summary to share at community engagement events. Additionally, we aim to produce a document identifying good practice in PPI for secondary data analysis to share with researchers.

Two limitations of the review are the scope of the review being restricted to three publication years (2014, 2019 and 2024) and to one journal (*The BMJ*). While this decision balances a comprehensive approach with pragmatism, since *The BMJ* is a journal with a wide scope and the years have been carefully selected to observe trends over time before and after PPI statements were mandatory, the generalisability of findings may still be limited and instead motivate future research across other years or in other journals where PPI statements may have become mandatory at different points in time. A further limitation is that we are adopting a qualitative descriptive approach to categorise and summarise PPI statements rather than more interpretative methods such as a thematic analysis [13, 14]. This choice is appropriate for our aims but does not provide deeper theoretical insights into PPI practices.

Ultimately, this review aims to promote more inclusive, transparent and impactful research by encouraging meaningful and context‐appropriate PPI in secondary data analysis. The involvement of public contributors in the review process itself further ensures that the findings and recommendations are grounded in lived experience and aligned with the values of co‐production, with joint decision‐making and genuine collaboration between researchers and the public.

## Author Contributions

L.G. was responsible for study conceptualisation. J.B., L.G., E.F., E.B. and J.B. were responsible for study methodology. J.B. was responsible for the original draft, and all authors were responsible for reviewing and editing the manuscript.

## Funding

The authors received no specific funding for this work.

## Ethics Statement

As this is a review of published literature, ethics approval/patient consent are not required.

## Conflicts of Interest

The authors declare no conflicts of interest.

## Supporting information

Example workbook.

## Data Availability

No new datasets will be generated. All relevant materials will be made available upon request.
